# Effect of extracorporeal shock wave therapy for rotator cuff tendonitis: A protocol for systematic review and meta-analysis

**DOI:** 10.1097/MD.0000000000022661

**Published:** 2020-11-25

**Authors:** Kewei Chen, Shuai Yin, Xiaodan Wang, Qianqian Lin, Huijie Duan, Zhenhua Zhang, Yiniu Chang, Yujing Gu, Mingli Wu, Nan Wu, Chengmei Liu

**Affiliations:** a Henan University of Chinese Medicine; b The First Affiliated Hospital of Henan University of Chinese Medicine, Zhengzhou, Henan, China.

**Keywords:** extracorporeal shock wave therapy, rotator cuff tendinitis, calcification, meta-analysis, protocol

## Abstract

**Background:**

Rotator cuff tendinitis is a highly prevalent cause of shoulder pain and leads to decreased patient quality of life. Extracorporeal shock wave therapy (ESWT) and ultrasound-guided needling are considered beneficial for rotator cuff tendinitis. A systematic review and meta-analysis comparing ESWT with sham-ESWT or ultrasound-guided needling in the management of pain and calcification is lacking.

**Methods:**

We will search the following up database from its inception to August 2020 without language restriction: PubMed, Cochrane Library, Web of Science, EMBASE, China National Knowledge Infrastructure, China Biomedical Literature Database, Chinese Science Journal Database, and WangFang database. All randomized controlled trials compared the effect of ESWT and sham-ESWT or ultrasound-guided needling of rotator cuff tendinitis will be included in pain and calcification. Two researchers will operate literature retrieval, screening, information extraction, quality assessment, and data analysis independently. The analysis will be conducted using Review Manager 5.3 Software.

**Results:**

The findings will be submitted to a peer-reviewed publication.

**Conclusion:**

This systematic review and meta-analysis will provide high-quality evidence for the treatment of patients with rotator cuff tendinitis.

**INPLASY registration number:**

INPLASY202080028

## 1 Introduction

Rotator cuff tendinitis is a common musculoskeletal disease and the leading cause of shoulder pain and movement disorders^[1]^; it occurs in an estimated 10% to 42% of the patients with shoulder complaints.^[2]^ Age, overuse, smoking, and obesity contribute to the disease.^[3,4]^ The reported prevalence of rotator cuff tendinitis varies from 2.7% to 20%, mostly affecting women aged 30 years.^[5,6]^ Yamaguchi et al^[7]^ found that the incidence rate among people over 50 years old was as high as 54%, and with the aging of the social population, the incidence rate presented an obvious rising trend.^[8,9]^ The main clinical manifestations of rotator cuff tendinitis are pain, shoulder dysfunction, hyperplasia, and deposits; these manifestations eventually cause progressive disability, psychosocial problems, and reduce patient quality of life.^[10]^ Research has shown that due to rotator cuff syndrome, 1.6 out of 1000 filed claims with the average cost of USD 21,872 and 260 lost days per compensable claim.^[11]^ This highly prevalent disease and the accompanying disability have terrible effects on individuals and society.

Currently, the treatment modalities for rotator cuff tendinitis remain controversial; it consists of surgery management, drug therapy, and nondrug therapy.^[12,13]^ Nonsurgical therapy is a first-line approach for rotator cuff tendinitis, with surgical management being reserved for those who fail a trial of conservative treatment.^[14]^ An initial conservative management plan of rotator cuff tendinitis includes adjusting the activity plan, self-management, and the use of simple analgesics if required.^[13]^ Drug management for rotator cuff tendinitis mainly consists of analgesics and nonsteroidal anti-inflammatory drugs. However, drug therapy may cause serious adverse gastrointestinal, renal, and cardiovascular events, which limits its application.^[15,16]^ Comparison with drug therapy, the guidelines for rotator cuff tendinitis are more recommend nonpharmaceutical methods.^[12,13]^

Over the past few decades, as a less invasive treatment, ultrasound-guided needling has been shown to be an effective second-line of rotator cuff tendinitis,^[17]^ but some problems remain, such as post-injection pain and bursitis, which commonly lead to second treatments.^[18]^ Extracorporeal shock wave therapy (ESWT), one of nondrug therapy, is a hot spot in clinical research. Its mechanism of action is that the shock wave is sent by the shock wave therapeutic apparatus through the positioning and movement of the treatment probe,^[19]^ which is transmitted to the pain point and produces mechanical stress and biological effects, thus promoting blood and lymphatic return, accelerating the metabolism, and inhibiting the high-frequency pulse from the pain medium and receptor.^[20,21]^ At present, ESWT has achieved significant results in the treatment of many musculoskeletal diseases, such as plantar fasciitis, knee osteoarthritis, tennis elbow, and rotator cuff injuries.^[22–25]^ A variety of trials have reported that ESWT is an effective method of dissolving calcification and stimulating tissue healing.^[26–28]^ As a noninvasive, safe, and easy physical therapy, ESWT provides a new therapeutic tool for orthopedics and rehabilitation as well as saves medical resources.

Despite several meta-analyses have been conducted to assess the efficiency of ESWT or ultrasound-guided needling of patients with rotator cuff tendinitis,^[17,28]^ there is no direct meta-analysis and systematic study of randomized controlled trials between ESWT and ultrasound-guided needling. Therefore, the aim of this systematic review and meta-analysis is to determine the effectiveness of ESWT for pain and calcification in patients with rotator cuff tendinitis, meanwhile, definite whether ESWT is more effective than ultrasound-guided needling.

## 2 Method and analysis

### 2.1 Study registration

The systematic review and meta-analysis has been registered at INPLASY (ID:INPLASY202080028). We will conduct this protocol following the guidelines of Cochrane Handbook for Systematic Reviews of Interventions and the Preferred Reporting Items to conduct this Systematic Reviews and Meta-analysis Protocol (PRISMA-P) statement.^[29]^

### 2.2 Eligibility criteria

#### 2.2.1 Types of study

This study will include only clinical randomized controlled trials. Case reports, nonrandomized clinical studies, quasi-RCTs, reviews, and animal studies will be excluded.

#### 2.2.2 Types of participants

Patients with clinically diagnosed of rotator cuff tendinitis will be fully considered for inclusion with any restrictions, such as race, age, and gender.

#### 2.2.3 Types of interventions

All patients in the experimental group must have received ESWT, while all subjects in the control group have accepted sham-ESWT or ultrasound-guided needling.

#### 2.2.4 Types of outcomes

Pain intensity and size of calcification are the primary outcomes. Pain intensity will be assessed by Visual Analogue Scale or Numerical Rating Scale. The size of calcification will be evaluated by radiographs or ultrasound imaging or Gärtner classification. Clinical outcomes, the function of shoulder, and adverse events are assumed as the secondary outcomes.

### 2.3 Search strategy

PubMed, Cochrane Library, Web of Science, EMBASE, China National Knowledge Infrastructure, China Biomedical Literature Database, Chinese Science Journal Database, and WangFang database will be searched without language restrictions. Studies will be searched up to the date the searches are run. We will also trace the references of relevant studies to ensure that any potential eligible RCTs will not be missed. The search strategy for PubMed is recorded in Table 1. Adapt to different Chinese and English databases, we will provide similar search strategies.

**Table 1:** The search strategy for PubMed.

Number	Search terms
1	Rotator cuff tendinitis
2	Rotator cuff
3	Shoulder
4	1 or 2–3
5	Extracorporeal shock wave therapy
6	Shock wave
7	Shockwave
8	Shockwaves
9	5 or 6–8
10	randomized controlled trial
11	Randomized
12	Placebo
13	10 or 11–12
14	4 and 9 and 13

### 2.4 Data collection and analysis

#### 2.4.1 Selection of studies

**Figure 1:**
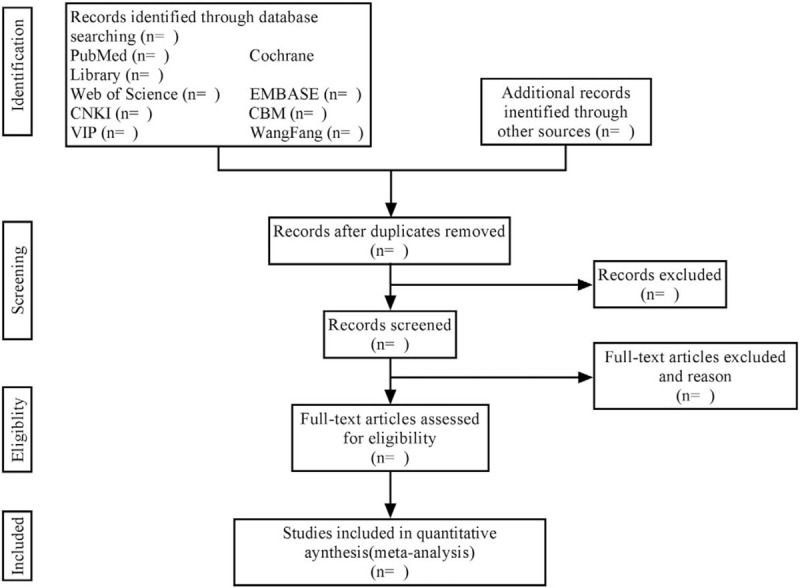
Flowchart of the study selection.

Two reviewers will independently search the literature according to the predetermined search strategy, the results of search will be imported into EndNote X9 software. After the elimination of duplicates, the 2 reviewers will identify the literature that may meet the eligibility criteria by browsing the titles and abstracts of the preliminarily screened literature. Then, by reading the full text of all eligible studies to decide whether a trial will be included. At the same time, the 2 reviewers will carry out cross-checking and extract relevant information and data. If there is any disagreement, discuss to solve the differences or consult the third reviewer. A flowchart (Fig. 1) will be used to depict the selection process of the study.

#### 2.4.2 Data extraction and management

The data extraction will be conducted by 2 reviewers, respectively. The following data will be extracted and recorded on the upfront excel sheet: the first author, publication year, country, sample size, treatment measures, study design and methods, assessment time, follow-up time, and outcome index. Any divergence will be resolved by discussion; if the results of the discussion cannot be unified, a third reviewer will be invited.

#### 2.4.3 Assessment of risk of bias

The quality evaluation of the included study will be conducted by 2 reviewers using Cochrane collaboration's tool, covering selection bias, performance bias, detection bias, attrition bias, reporting bias and other bias, each rated as low-risk, unclear and high-risk, and consulted a third reviewer when necessary. Two reviewers will complete the process independently and a third reviewer will be consulted if their evaluation results are inconsistent.

#### 2.4.4 Data analysis

When the data permits, the analysis will be done using Review Manager 5.3 Software. The heterogeneity between the included studies will be analyzed by Cochran's Q test and Higgins *I*^2^ statistic. If there is no statistical heterogeneity between the results of each study (*P* > .1, *I*^2^ < 50%), fixed-effect model will be used for meta-analysis. If not, the source of heterogeneity will be further analyzed and random-effect model will be used for analysis after excluding the influence of obvious clinical heterogeneity. All outcomes will be analyzed through 95% confidence intervals (95% CIs). If there are insufficient data to support meta-analysis, descriptive analysis will be implemented.

#### 2.4.5 Subgroup analysis

Subgroup analyses will be conducted for people with different control groups or different follow-up times when data are available.

#### 2.4.6 Sensitivity analysis

Sensitivity analysis is performed when Cochran's Q test and Higgins *I*^2^ statistic show significant heterogeneity. We will eliminate each study one by one to identify literature that have apparent impact on the original effects. The reasons for the obvious heterogeneity of the literature will be scrutinized from the aspects of sample size, study quality, and statistical methods.

#### 2.4.7 Assessment of reporting biases

If more than 10 articles will be included, a funnel plot would be used to demonstrate publication bias by its symmetry.

#### 2.4.8 Evaluation of the evidence quality

Two reviewers will respectively assess the evidence quality of each study by applying the Grading of Recommendations Assessment, Development and Evaluation system.^[30]^ The quality of evidence will be divided into high, moderate, low, and very low.

#### 2.4.9 Ethics and dissemination

Because this study does not involve private patient information, no ethical review is required. The results of this system review and meta-analysis will be published in a peer-reviewed journal.

## 3 Discussion

Rotator cuff tendinitis is a common cause of shoulder pain and functional decline, which has a great impact on patients’ lives and work to a certain extent. Therefore, effective exploration should be conducted in the management of rotator cuff tendinitis. As second-line treatment, ESWT and ultrasound-guided needling are widely used in the treatment of rotator cuff tendinitis. However, there is a short of a direct study comparing ESWT and ultrasound-guided needling in pain and calcification in patients with rotator cuff tendinitis. This systematic review and meta-analysis will summarize the most recent RCTs, to determine the effects of ESWT, compare ESWT and ultrasound-guided needling for rotator cuff tendinitis. We hope that this study will provide evidence for the clinical treatment of patients with rotator cuff tendinitis.

## Author contributions

**Conceptualization:** Kewei Chen, Shuai Yin, Xiaodan Wang.

**Data curation:** Qianqian Lin, Zhenhua Zhang.

**Formal analysis:** Kewei Chen, Xiaodan Wang.

**Funding acquisition:** Chengmei Liu, Nan Wu.

**Methodology:** Shuai Yin, Huijie Duan, Yiniu Chang.

**Project administration:** Chengmei Liu.

**Resources:** Yujing Gu, Mingli Wu.

**Software:** Qianqian Lin, Yiniu Chang.

**Supervision:** Chengmei Liu, Nan Wu.

**Writing – original draft:** Shuai Yin.

**Writing – review & editing:** Kewei Chen, Xiaodan Wang.
